# Changing the Organizational Structure and Enhancing Nurse-Staffing Levels Based on Patient Acuity in a Tertiary Referral Hospital

**DOI:** 10.1155/jonm/8894266

**Published:** 2025-10-28

**Authors:** Montserrat Martínez-Muñoz, Maria Angeles Barba-Flores, Dani Andrés Escobedo, Irene Joana Batuecas-Duelt, Sara López-Branchadell, Patricia Rubio-Garrido, Dimelza Osorio, Esperanza Zuriguel-Pérez

**Affiliations:** ^1^Health Services Research Group, Institut de Recerca Vall d'Hebron, Barcelona, Spain; ^2^Department of Medicine, Faculty of Medicine, Univ Autonoma Barcelona, Barcelona, Spain; ^3^Nursing Management, Department of Knowledge Management and Evaluation, Hospital Universitari Vall d'Hebron, Barcelona, Spain; ^4^Nursing Management, Hospital Universitari Vall d'Hebron, Barcelona, Spain; ^5^Multidisciplinary Nursing Research Group, Institut de Recerca Vall d'Hebron, Barcelona, Spain; ^6^Department of Knowledge Management and Evaluation, Hospital Universitari Vall d'Hebron, Barcelona, Spain; ^7^Centro de Investigación Biomédica en Red de Epidemiología y Salud Pública (CIBERESP), Madrid, Spain

**Keywords:** acuity management, nurse staffing, nursing intensity

## Abstract

**Aims:**

To enhance nurse-staffing levels in the wards of a tertiary referral hospital by applying a patient classification system based on patient acuity, setting up a new organizational structure in the hospital wards.

**Design:**

A retrospective analysis based on administrative data from an electronic patient database.

**Methods:**

The data were obtained from the clinical data warehouse of multiple databases from a tertiary referral hospital in Spain and analyzed from January 1st, 2018, to December 31st, 2019. The care plan and the weight of the main diagnosis from 52,974 adult patients admitted to 40 hospital units were analyzed and classified into patient acuity using the acute to intensive care (ATIC) classification system. The frequency of the ten acuity groups was analyzed, and the optimal patient-to-nurse ratio was defined according to the system and the hours effectively assigned to the care units. The percentage of patient needs that were met was also established. Finally, the distribution of the wards was rearranged based on the results.

**Results:**

The findings show that patients were mostly clustered in the following groups according to their acuity level: intensification (38.1%), intermediate (30.4%), and acute (21.8%). Therefore, the wards were reorganized into the three dominant levels: four acute, 17 intensification, and 19 intermediate units. A viable model was implemented to provide the number of nurse/patient/day hours by incorporating 104 nurses and improving nursing coverage by 17.4%.

**Conclusion:**

The structuring of care units according to the intensity of care has resulted in a significant enhancement in nursing coverage by improving the hours of care needed according to acuity levels.

**Implications for Patient Care:**

The findings provide valuable information regarding nurse staffing in hospitals, emphasizing the importance of defining the optimal patient-to-nurse ratio. Establishing this ratio is crucial for improving care quality and ensuring patient safety.

**Reporting Method:**

This study's methods and results have been reported following the STROBE checklist.

**Patient or Public Contributions:**

No patient or public contributed to design this research. However, patients will contribute later as part of a broader project when we explore their perception following the implementation of a management model based on nursing care intensity.

## 1. Introduction and Background

Healthcare management is rapidly evolving toward new, more sustainable organizational models, integrating a biopsychosocial approach to improve both health outcomes and patient experience. This transformation can be attributed, at least in part, to sociodemographic changes, including increased life expectancy, the prevalence of chronic diseases, and the increased fragility of populations, which are significantly impacting traditional healthcare models [1].

The patients seen in health services are becoming increasingly complex, often presenting with multiple comorbidities that increase their vulnerability. Conditions such as malnutrition, dementia, and diabetes are prevalent and can worsen during hospitalization. This highlights the growing need for nursing care. Future hospital strategy reports indicate that more specialized and differentiated care levels will be required to address the specific needs of patient care processes [2, 3].

Nursing care plays a crucial role in the hospitalization process and direct patient care. It is therefore reasonable to conclude that the effective management of nursing care will have a direct impact on health outcomes, the patient experience, and the quality and safety of care. Numerous studies support this assertion, for instance, the pioneering research of Aiken et al. [4, 5], which shows a direct relationship between the number of nurses, their level of education, and hospital mortality. Furthermore, these studies provide evidence of improvements in patient safety and care quality indicators, as well as in the prevention of complications and hospital readmissions. Other researchers have pointed out that better nursing staff adequacy can reduce professional burnout and turnover, which is critical given the international shortage of nurses [6]. Therefore, models that appropriately adjust the number of nurses per patient should be pursued to provide better care quality [7, 8].

One of these models is the All Patient Refined Diagnosis-Related Groups (APR-DRG), a widely used patient classification system. This system groups patients according to medical conditions and procedures and classifies them into four categories of severity and risk of mortality, ranging from low to extreme. However, the APR-DRG system does not take into account the variability in nursing care requirements [9].

Another model is the one proposed in the Canadian Project Research in Nursing (PRN, 1987). This system quantifies the time required for care actions from a procedural perspective. However, it does not include nurse-staffing focus on patient needs; therefore, it is insufficient to reflect the complexity of nursing care [10]. The PRN has been used in Spain to evaluate nursing workloads in over 100 hospital units. However, most results have not been published, as they form part of consultancy activities. For this reason, some researchers have proposed new formulas that consider variables such as nurse-sensitive outcomes [11], the influence of organizational context on care delivery [12], individual complexity factors [13, 14], or the predictive capacity of nursing diagnoses [15].

The acute to intensive care (ATIC) patient classification system is a validated tool for safety [15, 16]. It assesses acuity by assigning weight to the primary nursing diagnosis identified in the care plan. This tool provides us with the weights, already calculated based on a formula that considers severity and risk of death. The value of this weight indicates the level of intensity of care, which is translated into the number of nursing hours required per patient per day.

The ATIC system categorizes patients into ten acuity levels, ranging from “occasional” to “gigaintensive” care, allowing for precise stratification of care needs. This enables the estimation of staffing requirements by comparing the nursing hours each patient requires with the actual hours of care delivered.

Despite its strong predictive capability, the information generated by the system has not yet been widely leveraged to actively reorganize nursing resources according to patient acuity, nor to evaluate the impact of such redistribution on staffing adequacy across hospital units. However, previous studies using the ATIC system have already demonstrated an association between nurse staffing coverage and patient health outcomes, supporting its potential as a tool to optimize the quality and safety of care [17, 18].

In our hospital, a tertiary referral center in Spain, we historically have used the APR-DRG system to classify patients and manage nursing resources. In our experience, this approach was effective in achieving a homogeneous distribution of resources but did not comprehensively address potential inequities in resource allocation among different patients and hospital units.

In recent years, our hospital has experienced significant organizational evolution, transitioning from a traditional model based on specialized clinical services to an innovative interdisciplinary model. This new approach organizes our services and units around specific areas of knowledge and health problems, promoting more integrated and patient-centered care [19].

As part of this reorganization, we are implementing an optimized nurse-to-patient assignment model based on actual patient complexity. This study uses the ATIC system to readjust the distribution of nursing resources according to patient acuity and evaluates the impact of this redistribution on nurse staffing adequacy across hospital units.

## 2. Methods

### 2.1. Study Design

This study is the first phase of a larger project designed to evaluate a comprehensive model of care intensity management. For this study, we carried out a retrospective analysis based on records from an electronic patient database. Data were collected from January 1st, 2018, to December 31st, 2019, and accessed in 2021. We used the Strengthening the Reporting of Observational Studies in Epidemiology (STROBE) criteria [20] to report the findings.

### 2.2. Setting and Participants

The study was conducted at the Hospital Universitari Vall d'Hebron (HUVH) in Barcelona, Spain. This hospital has over 1000 beds, serves a reference population of more than half a million citizens, and employs around 9000 professionals, of whom one-third are nurses. The HUVH is comprised of four hospitals: General, Children's and Women's, and Trauma and Rehabilitation, and Burns. This tertiary-level hospital complex is part of the Catalan Institute of Health's public hospital network and of the Alliance of European University Hospitals (EUHA).

For the patient acuity analysis, we included all patients over 17.9 years old and admitted consecutively to any of the 40 hospitalization units of HUVH during the study period. Patients who were exclusively admitted to intensive care units (ICUs) were excluded from the study, as the objective of the study was to focus on conventional hospitalization units.

For this study, each stay in a hospital unit was considered a patient episode. Since patients can be transferred between different units, the total number of episodes at the unit level is expected to be higher than the total number of episodes at the hospital level. The initial estimated population was 52,974 patients.

For the nursing staffing levels measure, we considered the care nurses working in the study units. Nurse managers were excluded from the analysis. In HUVH, nurses use standardized care plans based on ATIC terminology [21]. These care plans, based on the reason for admission, can be adjusted by each nurse according to patient needs, based on their clinical judgment.

### 2.3. Data Collection

Two key indicators were collected to characterize nurse staffing: required nursing hours per patient per day (rNHPPD) and available nursing hours per patient per day (aNHPPD).

The rNHPPD was estimated based on the main nursing diagnosis documented in the electronic nursing records, using the ATIC classification system, which categorizes care intensity into ten levels. Each level corresponds to a specific number of nursing hours required per patient per day. For each patient, the average rNHPPD was calculated over the entire hospital stay.

Data were retrospectively retrieved from the hospital's clinical data warehouse, which contains anonymized electronic health records from multiple institutional databases. The collected variables included demographic information (e.g., age and gender), hospitalization unit, length of stay, and integrated knowledge areas. In addition, the nursing care plan and the weight of the main ATIC diagnosis were extracted from the electronic nursing records.

Information on staffing levels was obtained from the structural staffing allocation reports of each inpatient unit. These reports provided detailed data on the number of nursing hours available per patient per day, aggregated by unit, shift, and date. Based on this, a daily average aNHPPD was calculated for each unit.

Each patient was assigned a unique sequential identifier to ensure data traceability and organization. The quality and completeness of the collected data were assessed using a structured data collection sheet developed in Microsoft Excel.

### 2.4. Data Analysis

Data collected for analysis included the number of beds per inpatient unit (to estimate staffing needs), the nurse-to-patient ratio expressed as NHPPD, and the care intensity group based on patient profiles.

The analysis began with a theoretical model that defines the rNHPPD to ensure patient safety. This model, detailed in Table 1 under the concept of nursing presence excellence, establishes the optimal level of nurse staffing needed to meet care demands.

By comparing this reference model to actual staffing levels, it was possible to determine the gap between required and available nurse hours. The available nursing hours (aNHPPD) represent the actual nursing hours assigned per unit. A proportional adjustment model was developed to improve the balance between rNHPPD and aNHPPD, thereby providing a practical framework for aligning staffing levels with patient care needs.

The difference between rNHPPD and aNHPPD, expressed as a percentage, indicates the extent to which patient safety needs are met by the available nursing staff as shown in Table 2.

Qualitative variables expressed as frequencies and percentages, providing a clear view of the distribution of key characteristics. Quantitative variables, in turn, were summarized using means and ranges (minimum–maximum), which allowed for identifying patterns and variations in care intensity levels. All analyses were performed using SPSS software, Version 24.

### 2.5. Ethical Considerations

The research was approved by the hospital's Clinical Research Ethics Committee (reference 136/2023) and adhered to the principles outlined in the Declaration of Helsinki. The need for informed consent was waived due to the anonymous nature of the data. Additionally, the data were processed in compliance with relevant national legislation.

## 3. Results

During the study period, 52,974 patients were admitted to 40 hospital units, and 68,585 patient episodes were recorded. No patients were excluded due to missing data, nor were episodes with duplicated numbers excluded.

### 3.1. Patient Acuity

**Figure 1:**
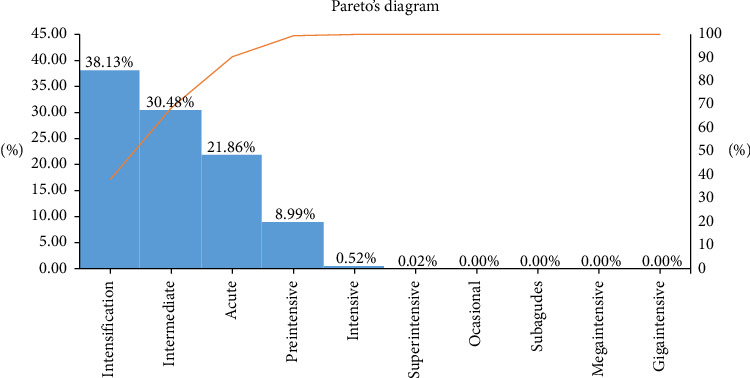
Care plans and correspondence with acuity groups.

Overall, 38.1% of patients presented an intensification acuity level, 30.4% an intermediate acuity level, 21.8% an acute care level, 8.9% had a preintensive level, and 0.5% an intensive level, while only 0.02% required superintensive level. No patients were found in the other acuity levels of the ATIC patient classification system.  Figure 1 shows the correspondence between the care plans used in the analysis and the acuity groups in a Pareto diagram.

We identified 12 prevalent care plans corresponding to the most frequent reasons for admission. The four main care plans were postpartum care for vaginal delivery (4.8%) and cesarean section (3.7%), both classified as intensification; short-stay post-surgical trauma care (4%), classified as acute; and acute stroke care (3.3%), classified as intermediate.  Table 3 details these care plans, their respective care intensity group, and the weight of the primary associated diagnosis.

**Figure 2:**
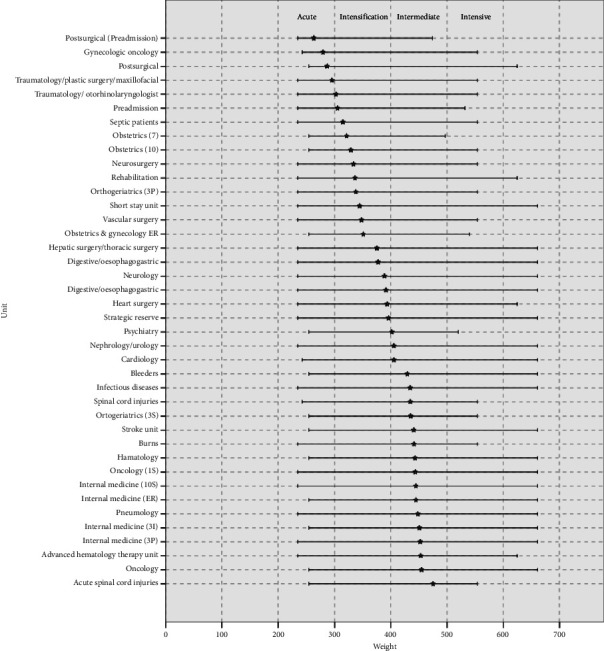
Distribution of the hospitalization units (*N* = 40) according to the level of intensity of care required.

As illustrated in Figure 2, a bar chart is employed to depict the distribution of hospitalization units according to the level of required care intensity. The data are grouped according to the ATIC classification, which defines the following ranges of primary nursing diagnosis weight: 201–300 for acute care, 301–400 for intensification care, and 401–500 for intermediate care. The graph shows the maximum, minimum, and average intensity values for each category.

As evidenced by the results, the care units have been classified into three levels of care intensity, as follows: four units (10%) with an acute care intensity profile, 17 units (42.5%) with an intensification care intensity profile, and 19 units (47.5%) with an intermediate care intensity profile.

Table 4 provides detailed information on the different care intensity levels for each hospitalization unit, as well as rNHPPD.

### 3.2. Nursing Staffing Measures

During the implementation of the new organizational model in our hospital—based on integrated knowledge areas and specific health problems—40 care units were reorganized into 20 integrated knowledge areas. Nursing staffing levels were adjusted according to measurements of patient care intensity. Table 1 compares staffing metrics across three different models: the theoretical model (based on excellence benchmarks from [15, 16]), the previous model (based on the APR-DRG system), and the viable model that was ultimately implemented, taking into account the attributes analyzed in this study.

When comparing the nurse-to-patient ratios from the theoretical model with the actual situation in adult inpatient units, daytime ratios ranged from 1:4 to 1:9, with an average of 1:7. For night shifts, ratios ranged from 1:10 to 1:15, averaging 1:12. To fully meet the theoretical model's standards across all units, an additional 271 nursing staff members would have been required.

Due to budget constraints, workforce shortages, and recruitment challenges, achieving this full increase was not feasible. Instead, a proportional staffing increase of 14.28%—based on the available resources—was approved. Through a more efficient organizational redesign and staff allocation aligned with patient care intensity, it was possible to hire 104 additional nurses. This staffing increase represented 38.37% of the total gap between the existing and the theoretically ideal staffing levels (104 out of the 271 required nurses).

Following implementation of the feasible model, a balance was analyzed between the required and aNHPPD. The coverage analysis showed that the gap between required NHPPD (rNHPPD) and actual NHPPD (aNHPPD) was reduced by 76.44%, which corresponds to a 17.44% improvement in nursing care hours per patient per day, as shown in Table 2.

## 4. Discussion

This study aimed to describe the acuity of patients admitted to hospital units and determine nurse staffing levels. The approach used in this study to calculate nurse staffing aligns with recent research that emphasizes the importance of including patient-related factors such as complexity or care intensity [15, 22, 23].

The results of this study reveal that a majority of hospitalized patients fall into intermediate and intensification acuity categories, confirming trends observed in previous research [15, 16]. At the hospital unit level, it was observed that patients admitted to medical units had the highest acuity profiles, followed by those hospitalized in surgical units. This finding is consistent with previous studies [16, 24]. However, in the context of task-centered care models, surgical units have been better staffed with nurses in response to the increasing complexity of procedures [25].

Several studies have identified the coexistence of different levels of care in hospitalized patients [16, 26]. This variability is especially notable in postoperative care. It is essential that postoperative care assignments align with patient acuity, as highlighted in the literature [27]. Based on these findings, our postoperative units have been structured into three care levels: acute, intermediate, and intensification. Patient admission is now based on acuity rather than clinical specialty, ensuring that each patient receives the appropriate level of care according to their health status.

In general, although 12-h shifts like those used in our hospital tend to distribute staff more equitably between day and night, nighttime staffing still features a lower nurse-to-patient ratio compared to daytime shifts. This traditional shift structure, with 60% of staff assigned during the day and 40% at night, is based on a general estimate of nurse activity over a shift cycle. However, reducing the number of nurses during night shifts could increase patient vulnerability regarding safety issues [28, 29]. In light of this evidence, we have adjusted the nurse distribution to 55% during the day and 45% at night.

The restructuring of the hospital's organizational model into patient-centered integrated knowledge areas has facilitated the application of a management model based on care intensity. Moreover, the incorporation of clinical management tools, such as the care intensity map, plays a crucial role by providing a clear picture of the acuity levels in each unit and enabling more effective resource management. Since the ATIC classification system is integrated into the electronic health record, nurses do not need to duplicate information to obtain acuity levels.

Implementing this model has involved significant challenges, representing a transformative innovation compared to traditional hospitalization approaches. The problem of nurse staffing allocation is particularly complex, as patient distribution can generate dissatisfaction among staff, especially when it is not consistent, objective, or quantifiable [30–32].

Although implementing an intensity-based care management model may initially involve an increase in budget for hiring and retaining staff, the benefits in terms of reducing adverse events and hospital stays can justify this investment. Furthermore, this model can improve overall care efficiency and alleviate negative aspects for nurses, such as emotional exhaustion and the intention to quit their job [33, 34].

Findings in the literature consistently highlight the importance of having standardized and valid patient classification systems [35]. In our case, we used a validated method for estimating and predicting the rNHPPD, based on the main nursing diagnosis weight in the care plan [15]. Although we did not achieve the theoretical maximum number of nurses required for each care unit as described by Juvé [16] due to hiring budget limitations, we believe that the use of this system has optimized resource allocation while facilitating a more balanced distribution of nursing hours among patients.

### 4.1. Strengths and Limitations

To the best of our knowledge, this is the first study to apply the ATIC patient classification system to determine nurse staffing based on acuity as a hospital management model. This research offers a new perspective on improving resource allocation, supported by a large sample of patients, which adds strength and relevance to the study's findings. However, there are some limitations to consider. The exclusion of the pediatric population is significant, as the ATIC system may not have the same predictive capacity in this patient group. Additionally, collecting data from a single hospital could limit the generalizability of the results to other settings.

### 4.2. Recommendations for Further Research

The objective of this study is to provide an overview of nurse staffing levels from a systemic perspective. However, our study design limits the ability to assess how these levels impact clinical care, patient experience, and health outcomes. Future studies are recommended to analyze specifically how nurse staffing levels influence patient health outcomes. It is also important to investigate patient and staff satisfaction with the implementation of this management model. It is crucial to continue to evaluate and adjust our strategies according to the needs of the environment and economic capabilities, with the goal of maintaining an appropriate balance between quality of care and available resources. In addition, further research should be conducted to determine the validity of the system in the pediatric population.

### 4.3. Implications for Policy, Clinical Practice, and Patient Care

This study is significant because it provides a foundation for adjusting nurse staffing in hospitals, which is crucial for improving patient outcomes. Defining the optimal nurse-to-patient ratio is essential to ensure the quality of care and patient safety. Therefore, it is critical that staffing levels are adapted to the specific needs of patients, considering acuity levels.

Our management model proposes an innovative approach to patient classification, where care and staffing are assigned based on nursing diagnoses rather than a fixed staffing number for each unit. This allows for more personalized care, optimized resource allocation, increased satisfaction for both patients and healthcare professionals, and improved operational efficiency in hospitals.

## 5. Conclusion

The structuring of care units according to the intensity of care has resulted in a significant improvement in nursing coverage. This approach provides a more efficient structure of professionals, avoiding the reliance on subjective methods and unstable staffing patterns with high turnover of less specialized personnel. While the results are promising, further cost-benefit studies are needed to confirm the effectiveness of this model.

In summary, this management model aligns with current trends in technological integration and patient-centered care. Tailoring nursing time to the specific needs of each patient contributes to improving health outcomes and ensuring quality and safety of care within resource planning models in hospital management.

## Figures and Tables

**Table 1:** Comparison of nursing staffing measures between the theoretical model, existing model, and implemented workable model.

Theoretical model							Existing model				Workable model			
Hospital	Hospitalization units/integrated knowledge areas (IKA)	N. of beds	Intensity cluster care^1^	Ratio/intensity^2^	Nursing presence excellence^3^		Nursing presence		Necessary nursing presence excellence		Increased real presence^4^		Ratio/intensity	
					Day	Night	Day	Night	Day	Night	Day	Night	Day	Night
General hospital	Neurosurgery	27	Intensification	1:6	5	5	4	3	1	2	0	0	1:6.75	1:9
	Neurology	27	Intensification	1:6	5	5	3	2	2	3	1	1	1:6.75	1:9
	Stroke	9	Intermediate	1:4	2	2	2	2	0	0	0	0	1:4.5	1:4.5
	**IKA neurosciences**	**63**			**12**	**12**	**9**	**7**	**3**	**5**	**1**	**1**	**1:6.3**	**1:7.8**
	General surgery—LCA—hepatobiliary, pancreatic and thoracic surgery	28	Intensification	1:6	5	5	4	3	1	2	0	0	1:7	1:9.3
		26	Intensification	1:6	4	4	3	2	1	2	1	1	1:6.5	1:8.7
		27	Intermediate	1:4	7	7	4.5	4	2.5	3	0.5	0	1:5.4	1:6.8
	Short surgical stay	25	Intensification	1:6	4	4	3	2	1	2	1	1	1:6.3	1:8.3
	**IKA postsurgical care**	**106**			**20**	**20**	**14.5**	**11**	**5.5**	**9**	**2.5**	**2**	**1:6.2**	**1:8.2**
	Gastroenterology—endocrine Hepatology	27	Intermediate	1:4	7	7	3	3	4	4	2	1	1:5.4	1:6.8
		27	Intermediate	1:4	7	7	3	2	4	5	2	2	1:5.4	1:6.8
	**IKA digestive diseases** ** **	**54**			**14**	**14**	**6**	**5**	**8**	**9**	**4**	**3**	**1:5.4**	**1:6.8**
	Hematology	28	Intermediate	1:4	7	7	4	3	2	3	1	1	1:5.6	1:7
	Oncology	26	Intermediate	1:4	6	6	4	3	3	4	1	1	1:5.2	1:6.5
	Advanced hematology therapy unit	12	Intermediate	1:4	3	3	3	3	0	0	0	0	1:4	1:4
	**IKA oncohematology**	**66**			**16**	**16**	**11**	**9**	**5**	**7**	**2**	**2**	**1:5.1**	**1:6**
	**IKA infectious diseases**	**18**	Intermediate	1:4	**4**	**4**	**2**	**2**	**2**	**2**	**1**	**0**	**1:6**	**1:9**
	**IKA mental health**	**8**	Intensification	1:6	**1**	**1**	**2**	**1**	**0**	**0**	**0**	**0**	**1:4**	**1:8**
	**IKA respiratory system**	**22**	Intermediate	1:4	**6**	**6**	**3**	**2**	**3**	**4**	**1**	**1**	**1:5.5**	**1:7.3**
	Nephrology	18	Intermediate	1:4	5	5	3	2	3	4	1	1	1:7	1:9.3
	Urology	10	Acute	1:8	1	1								
	**IKA renal system**	**28**			**6**	**6**	**3**	**2**	**3**	**4**	**1**	**1**	**1:7**	**1:9.3**
	**IKA solid organ transplant**	**28**	Intermediate	1:4	**7**	**7**	**4**	**3**	**3**	**4**	**1**	**1**	**1:5.6**	**1:7**
	Cardiology	14	Intermediate	1:4	3	3	3	2	2	3	1	1	1:6.8	1:9
		13	Acute	1:8	2	2								
	Heart surgery	27	Intermediate	1:4	7	7	3	2	4	5	2	2	1:5.4	1:6.8
	Coronary semicritical	4	Intermediate	1:4	1	1	1	1	0	0	0	0	1:4	1:4
	**IKA heart**	**58**			**13**	**13**	**7**	**5**	**6**	**8**	**3**	**3**	**1:5.8**	**1:7.3**
	Strategic reserve	**27**	Intermediate	1:4	**7**	**7**	**3**	**2**	**4**	**5**	**2**	**2**	**1:5.4**	**1:6.8**
	**IKA internal medicine**	**28**	Intermediate	1:4	**7**	**7**	**3**	**2**	**4**	**5**	**2**	**2**	**1:5.6**	**1:7**

Traumatology, rehabilitation & burns hospital	**IKA spinal cord injuries**	**20**	Intermediate	**1:4**	**5**	**5**	**3**	**2.5**	**2**	**2.5**	**1**	**1**	**1:5**	**1:6.6**
	Plastic surgery/Maxillofacial	24	Acute	1:8	3	3	3	2.5	0	0.5	0	0	1:8	1:12
	Surgical short stay	26	Acute	1:8	3	3	3	3	0	0	0	0	1:8.6	1:13
	Processes	24	Acute	1:8	3	3	3	2	0	1	0	0	1:8	1:12
	**IKA post-surgical care**	**74**			**9**	**9**	**9**	**7.5**	**0**	**1.5**	**0**	**0**	**1:8.2**	**1:12.3**
	**IKA infection, tumors and osteoarticular reconstruction**.	**26**	Intensification	**1:6**	**4**	**4**	**3**	**2**	**1**	**1**	**1**	**1**	**1:6.5**	**1:8.6**
	Orthogeriatrics	26	Intermediate	1:4	6	6	3	2	3	4	2	2	1:5.2	1:6.5
	Orthopedic surgery and traumatology	28	Intermediate	1:4	7	7	4	3	3	4	1	1	1:5.6	1:7
	**IKA traumatic frail patient**	**54**			**13**	**13**	**7**	**5**	**6**	**8**	**3**	**3**	**1:5**	**1:6.1**
	**IKA neurorehabilitation**	**24**	Intensification	**1:6**	**4**	**4**	**3**	**2**	**1**	**2**	**1**	**1**	**1:6**	**1:8**
	**IKA burn patient care**	**20**	Intermediate	**1:4**	**5**	**5**	**5**	**3**	**0**	**2**	**0**	**1**	**1:4**	**1:5**

Women hospital	Maternity obstetrics A	14	Intensification	1:6	2	2	2	2	0	0	0	0	1:7	1:7
	Maternity obstetrics B	20	Intensification	1:6	3	3	3	3	0	0	0	0	1:6.6	1:6.6
	**IKA pregnancy and puerperium**	**34**			**5**	**5**	**5**	**5**	**0**	**0**	**0**	**0**	**1:6.8**	**1:6.8**
	**IKA postsurgical care**	**16**	Acute	**1:8**	**2**	**2**	**2.5**	**2**	**0**	**0**	**0.5**	**0**	**1:5.3**	**1:8**

									**Total excellence**		**Total viability**			
									**135 ∗ 2 = 271**		**52 ∗ 2 = 104**			

*Note:* Bold text indicate each Integrated Knowledge Areas (IKA).

^1^Based on the weight of the principal diagnosis and its equivalence to care intensity groups and nursing hours required per patient day.

^2^Provisioning to ensure safe nursing care.

^3^Calculation according to number of beds.

^4^DI adequacy: acute 3/2; intensification 4/3; intermediate 5/4.

**Table 2:** Nursing staffing measures.

Staffing measures	Preadequacy	Postadjustment	Difference
Median balance^a^	2.1	1.24	−0.86
Nursing coverage, mean %^b^	59%	76.44%	+17.44%

^a,b^Required: Average number of nursing hours per patient day required. Offered: Average number of nursing hours per patient day offered.

**Table 3:** Ranking of care plans, 2018–2019.

Care plan^a^	*N*	%	Weight of the main nursing diagnosis^b^	Intensity cluster care^c^
Vaginal delivery/puerperium	4103	4.8	301	Intensification
Short-stay post-surgical trauma care	3401	4.0	255	Acute
Caesarean section/puerperium	3180	3.7	359	Intensification
Stroke_acute phase	2794	3.3	418	Intermediate
Prosthetic joint surgery	2458	2.9	255	Acute
Endourological surgery	2271	2.1	359	Intensification
Infectious disease with pulmonary involvement	1995	2.3	453	Intermediate
Surgical fracture stabilization/bone resection	1748	2	301	Intensification
Urological post-surgical care	1732	2	255	Acute
Ischemic heart disease	1678	2	371	Intensification
Postoperative care of patients with intestinal disorders	1490	1.7	359	Intensification
Nonsurgical fracture stabilization	1471	1.7	359	Intensification

^a^A care plan using the ATIC terminology.

^b^The weight of the main nursing diagnosis represents a calculated value that indicates the intensity of care required. This value is determined based on the severity of the patient's condition, including the presence of comorbidities and the risk of mortality. The main nursing diagnosis is the one that generates the greatest need for care within the care plan.

^c^The intensity cluster care, as defined by the acute-to-intensive care (ATIC) patient classification system, identifies ten levels of intensity of care, each comprising 100 intensity points.

**Table 4:** Distribution of levels of intensity of care across inpatient units (*N* = 40).

Hospitalization units	N. beds	N. care plans	% Intensity cluster care				Primary issue weight			Intensity cluster care	NHPPD^∗∗^	More registered care plan^∗∗∗^
General hospital			Acute	Intensified	Intermediate^∗^	Intensive	Average	Mín.	Max.			
1P Internal medicine	10	659	0.9%	12.2%	86.6%	0.3%	444.69	255	661	Intermediate	6 h	Infectious diseases with lung involvement
1S Oncology	17	1441	3.1%	17.9%	78.5%	0.5%	443.49	235	661	Intermediate	6 h	Deterioration of the oncology patient's general condition
2P Cardiology	27	3575	13.8%	37.1%	49.0%	0.1%	405.51	243	661	Intermediate	5.5 h	Ischemic cardiomyopathy
2S Cardiac surgery	27	2697	14.5%	18.9%	66.5%	0.1%	393.51	235	625	Intensification	5 h	Cardiac surgery
P2 Center. stroke	9	2338	4.1%	3.7%	92.1%	0.1%	440.9	255	661	Intermediate	6 h	Stroke_ acute stage
3P Internal medicine	28	1426	1.8%	15.6%	81.6%	1.0%	452.4	235	661	Intermediate	6.5 h	Infectious diseases with lung involvement
3S Internal medicine	26	1359	1.5%	17.9%	80.4%	0.2%	450.78	255	661	Intermediate	6 h	Infectious diseases with lung involvement
4P Digestive/oesophagogastric	28	2500	9.6%	57.3%	27.8%	5.3%	377.37	235	661	Intensification	5 h	Thoracic surgery
4S Short-term unit	25	2974	23.8%	58.6%	17.4%	0.2%	344.49	235	661	Intensification	4 h	Post-surgical care of patients with intestinal disorders
5P Pneumology	27	2373	2.8%	17.2%	78.8%	1.2%	448.24	235	661	Intermediate	6 h	Infectious diseases with lung involvement
5S Vascular surgery	27	2126	26.0%	51.2%	22.8%	0.0%	347.87	235	554	Intensification	4 h	Peripheral ischemic complications
6P Nephrology/urology	28	2286	15.7%	20.1%	63.7%	1.3%	405.46	235	661	Intermediate	5.5 h	Renal function impairment study
6S Infectious diseases	18	1516	2.3%	17.4%	79.6%	0.7%	434.46	235	661	Intermediate	6 h	Infectious diseases with lung involvement
6S Psychiatry	8	373	1.1%	33.5%	65.4%	0.0%	401.98	255	520	Intermediate	5.5 h	Substance use disorder
6C Advanced hematology therapy unit	12	135	0.7%	28.1%	59.3%	11.9%	453.23	235	625	Intermediate	6.5 h	Systemic treatment with antineoplastic drugs
7P Oncology	26	2024	0.6%	14.5%	84.2%	0.7%	454.71	255	661	Intermediate	6.5 h	Deterioration of the oncology patient's general condition
7S Hematology	22	1674	0.7%	28.4%	65.9%	5.0%	443.03	255	661	Intermediate	6 h	Systemic treatment with antineoplastic drugs
7Annex. preadmission	10	3048	49.6%	47.3%	3.1%	0.0%	305.19	235	532	Intensification	3.5 h	Endourological surgery
8P Hepatic surgery/thoracic surgery	27	3034	19.5%	39.0%	39.2%	2.3%	375.13	235	661	Intensification	5 h	Endourological surgery
8S Digestive/endocrinology	24	2421	1.9%	41.0%	57.1%	0.0%	391.37	235	661	Intensification	5 h	Patient's care with with gastrointestinal bleeding_Stable stage
8 Bleeders	4	936	0.2%	2.9%	96.7%	0.2%	429.43	255	661	Intermediate	6 h	Patient's care with with gastrointestinal bleeding_Stable stage
9P Neurosurgery	27	1700	21.8%	64.8%	13.4%	0.0%	333.52	235	554	Intensification	4 h	Craniotomy
9S Neurology	28	2556	4.6%	42.3%	53.1%	0.0%	388.67	235	661	Intensification	5 h	Stroke_ acute stage
10P Strategic reserve	27	2363	16.7%	29.1%	53.9%	0.3%	395.93	235	661	Intensification	5 h	Urological post-surgical care
10S Internal Medicine	28	1906	2.0%	23.5%	73.9%	0.6%	444.63	235	661	Intermediate	6 h	Hepatic encephalopathy

*Traumatology, rehabilitation & burns hospital*												
Burns	20	980	4.4%	2.3%	93.3%	0.0%	441.22	235	554	Intermediate	6 h	Post-surgical care
1P Spinal cord injuries	16	295	13.9%	6.4%	79.7%	0.0%	434.69	243	554	Intermediate	6 h	Complex chronic lesions in the spinal cord injured patient
1P Acute spinal cord injuries	4	111	7.2%	6.3%	86.5%	0.0%	475.06	255	554	Intermediate	6.5 h	Spinal cord injured patient unstable stage
1S Traumatology/plastic surgery/maxillofacial	24	2927	58.3%	34.1%	7.6%	0.0%	295.36	235	554	Acute	3 h	Oral surgery
2P Traumatology/otorhinolaryngologist	26	2277	55.2%	36.1%	8.7%	0.0%	302.39	235	554	Intensification	3.5 h	Post-surgical trauma care_short stay
2S Septic	26	1676	31.4%	57.3%	11.3%	0.0%	311.51	235	554	Intensification	3.5 h	Post-surgical care of the patient with osteoarticular infection
3P Traumatology	26	2651	32.9%	45.5%	21.6%	0.0%	337.75	235	554	Intensification	4 h	Post-surgical trauma care_short stay
3S Traumatology	28	2781	5.5%	24.7%	69.8%	0.0%	435.28	255	554	Intermediate	6 h	Femur surgery in frail patient
4P Rehabilitation	24	1023	29.3%	46.6%	23.9%	0.2%	336.27	235	625	Intensification	4 h	Stroke_intensive rehabilitation
4S Processes	24	2716	71.1%	21.7%	7.2%	0.0%	286.79	255	625	Acute	3 h	Prosthetic joint surgery
Preadmission		2792	87.0%	11.8%	1.2%	0.0%	263.50	235	474	Acute	2.75 h	Post-surgical trauma care_short stay

*Women's hospital*												
Obstetrics & Gynecology ER. Room B	29	3279	9.5%	74.1%	16.4%	0.0%	350.97	255	540	Intensification	4 h	Caesarean section/puerperium
P7 Obstetrics	21	3503	0.1%	95.8%	4.1%	0.0%	321.37	255	497	Intensification	3.5 h	Vaginal delivery/puerperium
P9 Onco-gynecology	26	2817	78.8%	14.2%	7.0%	0.0%	279.79	243	554	Acute	3 h	Gynecological post-surgical care
P10 Obstetrics	26	4582	8.6%	79.8%	11.6%	0.0%	328.83	255	554	Intensification	3.5 h	Vaginal delivery/puerperium

^∗^Intermediate + preintensives.

^∗∗^Nursing hours per patient per day according to acute to intensive care (ATIC) patient classification system.

^∗∗∗^Care plan using ATIC terminology.

## Data Availability

Data are available on request from the authors.
